# Recent Advances in Positive Photoresists: Mechanisms and Fabrication

**DOI:** 10.3390/ma17112552

**Published:** 2024-05-25

**Authors:** Muhammad Hassaan, Umama Saleem, Akash Singh, Abrar Jawad Haque, Kaiying Wang

**Affiliations:** Department of Microsystems, University of South-Eastern Norway, 3184 Horten, Norway; muhammadhassaan@ieee.org (M.H.); umamasaleem2013@gmail.com (U.S.); akashsinghchanchal@gmail.com (A.S.); zdhaque@gmail.com (A.J.H.)

**Keywords:** deep ultraviolet lithography, extreme ultraviolet lithography, chemically amplified resists, non-chemically amplified resists, organic resists, inorganic resists, hybrid photoresists, dry film photoresist

## Abstract

Photoresists are fundamental materials in photolithography and are crucial for precise patterning in microelectronic devices, MEMS, and nanostructures. This paper provides an in-depth review of recent advancements in positive photoresist research and development, focusing on discussion regarding the underlying mechanisms governing their behavior, exploring innovative fabrication techniques, and highlighting the advantages of the photoresist classes discussed. The paper begins by discussing the need for the development of new photoresist technologies, highlighting issues associated with adopting extreme ultraviolet photolithography and addressing these challenges through the development of advanced positive-tone resist materials with improved patterning features, resolution, and sensitivity. Subsequently, it discusses the working mechanisms and synthesis methods of different types and subtypes of photoresists, starting from non-chemically amplified, organic, and inorganic–organic hybrid photoresists and progressing to dry film resists, with an emphasis on the upsides of each. The paper concludes by discussing how future research in the field of lithography—prioritizing concerns related to environmental impacts, improved photoresist material and properties, and utilization of advanced quantum technology—can assist with revolutionizing lithography techniques.

## 1. Introduction

Over the past few decades, the density of electronic Integrated Circuits (ICs) has doubled every two years [1]. This has led to the production of smaller, faster, and more cost-effective computing devices. However, the manufacturing process of these microchips as well as that of micro-electromechanical and nanotechnology systems involves lithography, which is a crucial step that utilizes photoresists and influences the feature size of the silicon dies. These photoresists are chemical entities, either molecular or macromolecular, possessing high photosensitivity. Depending on the solubility the photoresists develop upon exposure to radiation, they are divided into either positive or negative tone resists.

Initially, negative resists held dominance in the semiconductor industry until the 1970s, primarily due to the perceived drawbacks of positive resists, such as their excessive cost and poor adhesion properties [2]. However, as device sizes continue to shrink in line with miniaturization, positive photoresists appear to offer better results, leading to their prominence in the industry [2]. Positive photoresists offer several key benefits, including superior resolution, high contrast, enhanced etch resistance, absence of swelling behavior during development, better edge resolution, and improved aspect ratios of structures [3].

In photolithography, the dimension limit of a patterned feature structure is governed by the wavelength of light radiation [4], rendering Moore’s law invalid. To design feature sizes with diameters of 20 nm and smaller in the semiconductor sector and next-generation lithography, modifications to the light source were necessary, and extreme ultraviolet (EUV) radiation at 13.5 nm has become suitable for that [5], replacing the deep ultraviolet (DUV) wavelength that operates at 193 nm. This further necessitated the change in the photoresist to align it with EUV lithography technology. Since this technology is aimed at a really small feature size, a thinner resist film was required compared to the earlier generations of photoresists since the resist film’s thickness is directly correlated with the aspect ratio of the patterning structure. As a consequence, it is desired for the photoresists to have higher absorbance and sensitivity to EUV light, mainly because of the low power and shorter wavelength of EUV light’s photons, which corresponds to higher energy—precisely, 14.3 times more—and a lower number of EUV photons than that of DUV photons [6]. Additionally, EUV-based photolithography has been identified by the International Technology Roadmap for Semiconductors (ITRS) as a promising technological option to fabricate under-10 nm half-pitch nodes. Such sustained progress in the manufacturing of nanoscale devices relies on enhancements to optical lithography’s patterning capabilities.

In the semiconductor industry, chemically amplified resists (CARs) have long been the dominant choice for chip fabrication. However, a major shift has occurred recently due to the recognition that CARs are nearing their resolution threshold [7], and because they mostly consist of low optically dense carbon, hydrogen, and oxygen, they are too transparent to be used for EUV technology [8]. While the ultimate goal has always been to develop resists with magnificent resolution, high sensitivity, and enhanced line width roughness simultaneously, the most reported resists have compromised on at least one of these properties, even if the others were successfully achieved. This trade-off among parameters highlights the primary concern in resist development.

Considering the substantial market demand in the semiconductor industry and the limitations of CARs in realizing 20 nm features for ultra-sensitive ICs, the focus has recently shifted towards non-chemically amplified resists (n-CARs) for next-generation photolithography [3]. They offer potential advantages for patterning structures with better line edge roughness (LER) and line width roughness (LWR) [4].

Currently, the development of these photoresists presents a crucial challenge and necessitates taking into account multiple photochemical parameters, since n-CARs allow access to 20 nm half-pitch nodes [9]. The size and composition of element molecules in EUV photoresists are important factors that affect pattern structure, sensitivity, and dosage.

Since the photon density of an EUV beam is typically low, the formation of secondary electrons leads to bond fission and the generation of radical molecules. Consequently, the reduced photon count in EUV, which is over ten times less than that of DUV for the same power, results in photon-shot-noise-induced roughness in extremely small line patterns (<10 nm) [10].

Recently, there has been notable progress in the development of organic and inorganic resists, followed by organometallic (organic–inorganic hybrid) photoresists that include nanoparticles and organometallic compounds as effective materials for EUV photoresists [11]. These hybrid resists exhibit a higher tendency to absorb EUV compared to n-CARs, i.e., approximately 4–5 times more [12,13]; thereby, the photon shot noise leads to higher sensitivity and reduced roughness of patterned features. The metals react to EUV with high quantum efficiency because of their exceptional redox properties [14], which further enhances the effectiveness of metal-containing photoresists in EUV lithography applications.

While the existing literature predominantly delves into specific aspects of positive photoresist development, this review paper uniquely focuses on addressing a paramount concern in contemporary semiconductor manufacturing: node reduction for chip and system-on-chip (SoC) design. By considering the advancements in positive photoresists within the framework of Moore’s law, and through a holistic examination of node scaling challenges and the evolving landscape of semiconductor technology, this paper aims to provide a comprehensive understanding of the critical role played by photoresist technology and the strategies for overcoming obstacles and propelling innovation in driving advancements in chip design. In Section 2, the paper discusses the working mechanisms and synthesis methods of various photoresists, beginning with n-CARs and organic up to inorganic–organic hybrid photoresists in their respective subsections. Subsequently, the discussion extends to dry film resists: evaluating their advantages and limitations as alternatives to liquid resists. Concluding the paper, Section 3 comprehensively summarizes the photoresist types discussed and provides a future framework and research directions for positive-tone resists.

## 2. Working Mechanism and Synthesis Methods

### 2.1. Non-Chemically Amplified Resists

Non-chemically amplified resists (n-CARs) are different from CARs in their polarity switching mechanism [15] under exposure to photons or electron beams. While CARs rely on external photoacid generators (PAGs) [16] and radiation or light to induce polarity changes between exposed and unexposed regions, n-CARs offer a blend of their resist backbone with photosensitive functionality [15,17] and undergo polarity shifts without any catalytic chemical amplification. Consequently, n-CAR formulations are notably less complex and solve the problem of acid diffusion, leading to enhanced LWR and LER [18]. These materials can exist in the form of either organic or molecular resists, each exhibiting distinct mechanisms of decomposition. Further, n-CARs typically incorporate light-sensitive functionalities into their chemical structure [3], which undergoes photo-degradation upon exposure to radiation or suitable light energy.

#### 2.1.1. Non-Chemically Amplified Polymeric Photoresist

**Figure 1:**
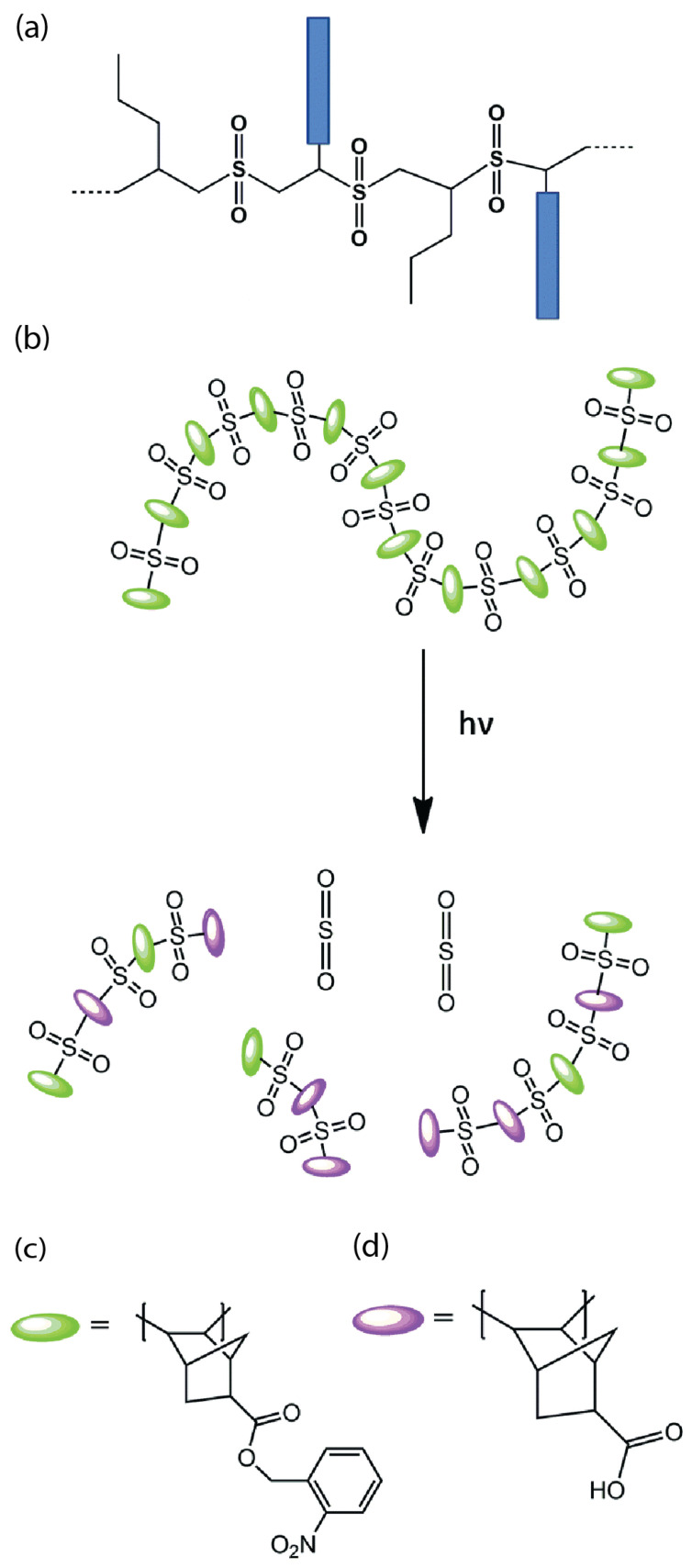
(**a**) Sulfone-based positive photoresist structure with poly(methyl methacrylate) arms (blue rectangles) [17]; (**b**) poly(olefin sulfone)-based photoresist undergoing solubility and molecular weight transition [3]; (**c**,**d**) o-nitrobenzyl and exo-5 norbornene derivatives, respectively [21].

For nanofeature lithography, sulfone-based resists are extensively utilized as non-chemically amplified chain degradation resists because of their high sensitivity towards many next-generation lithography techniques [19]. These formulations usually involve the fusion of a poly(olefin sulfone) backbone and poly(methyl methacrylate) (PMMA) arms, shown as blue rectangles in Figure 1a, forming ester groups with a comb-like structure. Upon EUV exposure, the backbone undergoes chain scission, which breaks the polymer chain into small fragments and allows for selective etching of the desired pattern. Whereas PMMA arms remain largely intact due to their high resistance and robustness and create a residual polymer network that provides structural support to the patterned features.

The hydrophobic functionality of these ester groups forms carboxylic acid groups, leading to consequent polarity alterations [20]. This transformation arises from the incorporation of the 2-nitrobenzyl bicyclo[2.2.1]hept-5-ene-2-carboxylate (NBHC) repeat unit (Figure 1c), which contains an o-nitrobenzyl group. This group undergoes photochemical cleavage upon photon exposure, generating a carboxylic acid. Notably, the norbornene component within this repeat unit alternates in the polymerization process with SO_2_, and this unique bicyclic structure confers enhanced etch resistance to the final polymer. Furthermore, photo-induced chain scission within the poly(olefin sulfone) backbone triggers depolymerization, resulting in a transition in the molecular weight (Figure 1d).

Since higher-molecular-weight resists offer reduced lateral diffusion, enhanced robustness, and higher sensitivity to light exposure, this transition results in improved contrast between exposed and unexposed regions, allowing for precise feature patterning with sharper edges. Furthermore, these resists often demonstrate increased resistance to etching processes, preserving the integrity of the patterned features and ensuring accurate reproduction of desired structures because of a stable and supportive network of PMMA arms.

#### 2.1.2. Non-Chemically Amplified Molecular Photoresist

**Figure 2:**
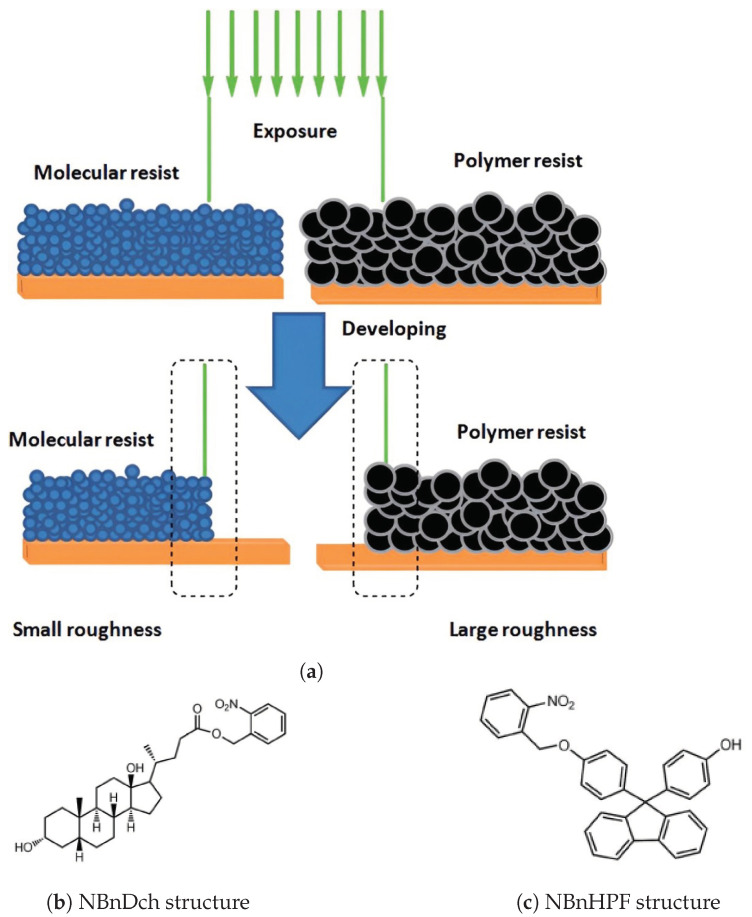
(**a**) Comparison of molecular and polymer resists for patterning techniques; (**b**,**c**) molecular n-CARs structures [3].

Most existing resist materials are polymer-based, primarily due to their amorphous nature, flexibility, and processability. However, their large molecular size presents a limitation [22], particularly affecting the LER of developed patterns, especially for nodes smaller than 20 nm. Each node width represents five to six polymer molecules, each ranging from 3 to 5 nm in size [3]. Consequently, polymer resists have inferior resolution and LER compared to those of molecular resists (Figure 2a).

Moreover, molecular resists prevent internal stress or swelling caused by intermolecular chain entanglement, thereby mitigating pattern distortion to a greater extent. Additionally, molecular resists made of similar molecules facilitate smooth and homogeneous thin film formation on suitable lithography substrates. These nitrobenzene-based resists incorporate nitrobenzyl as a photosensitive protection group in their molecular structure [3].

Upon exposure to light, this protective group undergoes deprotection, converting the inhibitory chemical compound responsible for dissolution into a promoter. As a result, exposed areas dissolve in a polar developer and convert these resists into molecular resists with a positive tone. Specifically, NBnDch (Figure 2b) utilizes deoxycholic acid as its base, while NBnHPF (Figure 2c) synthesizes on an aromatic platform featuring two phenolic groups. This results in the disintegration of exposed portions in a polar developer, leading to the positive tone of these resists in molecular forms.

The benefits of these molecular resists lead to the advancement in the development of high-performance lithographic techniques. Furthermore, the incorporation of specific molecular structures, like nitrobenzyl groups, offers tailored solutions for enhancing resist performance and enabling finer feature patterning at nanoscale dimensions. As semiconductor technology continues to push the boundaries of miniaturization, the evolution of molecular resists represents a pivotal step towards realizing high-density integrated circuits and advanced MEMS devices.

### 2.2. Organic Photoresists

Organic resists are composed of carbon-based compounds, such as polymers or small molecules, and are widely used in photolithography processes for semiconductor fabrication [23]. In general organic photoresists offer heightened sensitivity, tailored customization, superior etching selectivity, improved environmental compatibility, and reduced processing complexity, making them preferred for semiconductor and microfabrication applications [24]. Mono-substituted hydroquinone calix[8]arene and diazonaphthoquinone photoresists are good examples of organic photoresists. Here, macrocyclic compounds called calixarenes are well-known for their capacity to store other species inside their cavities. In the synthesis process of these calixarenes, one of their aromatic rings is swapped out for a single functional group, creating a mono-substituted derivative. When diazonaphthoquinone is added, the resulting chemical exhibits photosensitivity, which is necessary for patterning in microfabrication procedures [24].

Tomonari and his team investigate the possibility of utilizing the mono-substituted calix[8]arene [25], which is composed of m,m-methylene bridges and combines mono-substituted hydroquinone moieties, as an amorphous molecular material. This photoresist gives exceptional results in terms of improved dissolution characteristics, enhanced sensitivity, and being environmentally friendly [25]. Because of the outward-facing phenolic hydroxy groups in the planar model, the distinct structure of calixarenes implies potential dissolving properties for DNQ (diazonaphthoquinone) resist applications with high resolutions.

**Figure 3:**
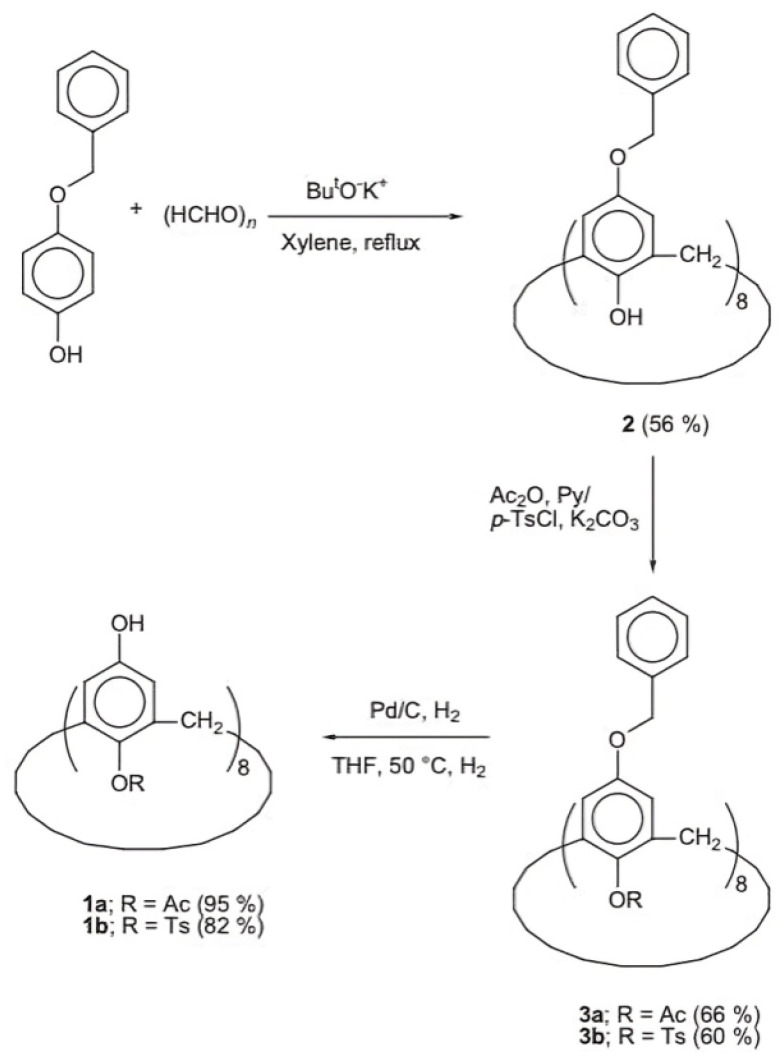
The synthesis scheme illustrates the stepwise process for generating O-substituted hydroxyphenol calix[8]arenes: **1a**: The preparation of p-(mono-acetyl)-calix[8]arene with a yield of 95%. **1b**: The preparation of p-(p-tolylsulfonyl)-calix[8]arene with a yield of 82%. **2**: The synthesis of p-(benzyloxy)-calix[8]arene. **3a**: Acetylation of Compound **2**, resulting in p-(mono-acetyl)-calix[8]arene with a yield of 66%. **3b**: Reaction of Compound **2** with toluene-p-sulfonyl chloride, yielding p-(p-tolylsulfonyl)-calix[8]arene with a yield of 60% [25].

**Figure 4:**
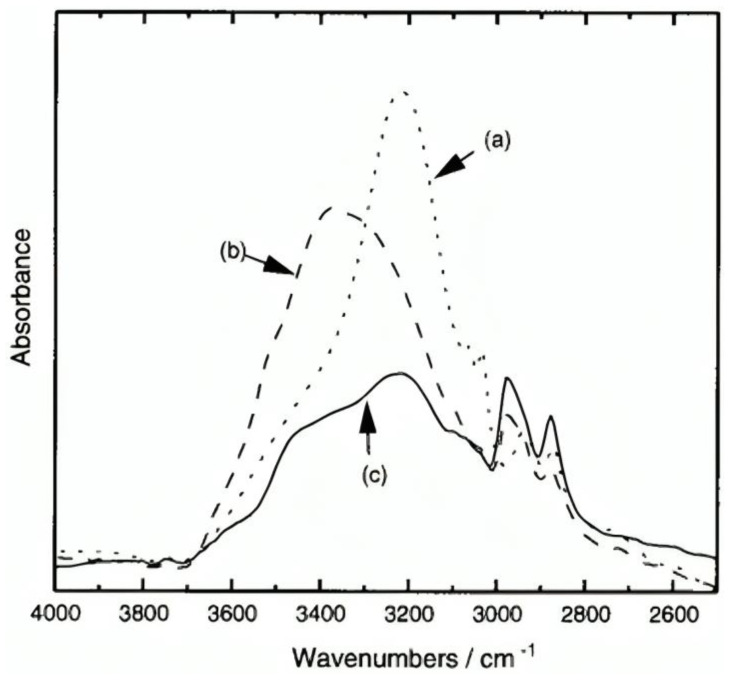
Infrared spectra of calixarenes: (**a**) **2** in a KBr pellet; (**b**) **la** and (**c**) **1b** films fabricated from THF on a NaCl plate [25].

The formation of mono-substituted hydroquinone calix[8]arenes involved various steps (Figure 3), starting with the preparation of p-(benzyloxy)-calix[8]arene (2) via base-catalyzed condensation with potassium tert-butoxide. Further acetylation and toluene-p-sulfonylation reactions were carried out to yield products **3a** and **3b**, respectively. Debenzylation of O-substituted-p-benzyloxyphenol calix[8]arenes was achieved using Pd/C under H2, yielding compounds **la** and **1b** [26]. These products exhibited high solubility in various polar solvents, facilitated by modifications enhancing intermolecular hydrogen bonding. This increased solubility enabled the formation of transparent films, demonstrating their potential utility in various applications requiring solubility and film formation properties [27]. In addition, (Figure 4) compares the IR spectra of three compounds: **2**, **1a**, and **1b**. A strong absorption band at 3220 cm^−1^ due to intramolecular hydrogen bonding can be seen in Compound **2**. In contrast, **1a** exhibits intermolecular hydrogen bonding, indicated by an absorption band at 3360 cm^−1^. Compound **1b** displays both types of hydrogen bonding. These modifications enhance solubility, making **1a** and **1b** soluble in various polar solvents. These can form highly transparent films when dissolved in solvents like ethyl lactate (EL) or (acetoxy-2-methoxyethane)PMA [25].

Typical components of organic photoresists include long chains made of synthetic polymer, which frequently contain aromatic rings present in materials such as acrylic derivatives and novolaks. These have light-responsive groups that are linked to the main polymer chain, and they react with specific wavelengths of radiation, causing active sites. The majority of commonly used organic photoresists can be classified as either photocrosslinking, photodecomposing, or photopolymerizing types [28].

The discovery of a new kind of positive-type photoresist using mono-substituted hydroquinone calix[8]arene marks a promising advancement in lithography technology. Researchers acquired exceptional sensitivity and contrast levels by combining calix[8]arene **1b** with DNQ(4) or DNQ(5), paving the way to high-quality envision reproduction. Calixarenes’ transparency makes them appealing choices for KrF excimer–laser lithography and allows them to exhibit versatility in a wide range of exposure techniques [29]. Overall, this breakthrough holds substantial potential for enhancing semiconductor manufacturing procedures and has applications in a variety of sectors.

Organic photoresists do not absorb light very well from advanced lithography tools like EUV, and they do not react well when used for etching, especially in dry conditions [24], but they exhibit enhanced sensitivity and resolution, which are crucial for advanced lithography techniques. In the case of dry etching, e.g., plasma etching, inorganic photoresists are more prone to etching. Just a thin layer of an inorganic photoresist is enough for protecting etching and ion implantation of underlying materials. Despite having a slightly higher aspect ratio, the use of inorganic photoresists has the additional benefit of making pattern collapse more difficult because it is more stable. Overall, organic photoresists often demonstrate improved environmental compatibility and reduced processing complexity, contributing to more sustainable and cost-effective manufacturing processes [25].

### 2.3. Inorganic–Organic Hybrid Photoresists

Recognizing the limitations of traditional photoresists for achieving sub-10 nm node widths and transparency of CARs to EUV because of less-optically dense atoms [30], organometallic resists are promising alternatives in advanced applications [31]. These EUV organometallic photoresists, comprising a combination of organic and inorganic elements, harness the benefits of both components since they use high-optically dense elements, particularly metals, and are classified as either metal nanoparticles or coordinative small molecules [5]. The inorganic constituent enhances the photosensitivity because of its higher tendency to absorb EUV light, strengthens the mechanical structure, leading to reduced pattern collapse, and provides better etch-resistance, hence, allowing for higher aspect ratios [32]. The organic component governs the elastic and ductile properties of the photoresist, allowing for crack-free coatings [33] and a change in solubility properties of the photoresist during development conditions.

#### 2.3.1. Metal Nanoparticles

Metal nanoparticles, typically 2–3 nm, emerge as an ideal candidate for advanced photoresist materials [34] and are capable of achieving under-10 nm half-pitch lines. They are structured so that they feature a single inorganic metal core surrounded by an outer shell made up of organic ligands, and they offer unique redox properties that could readily respond to EUV radiation.

The photolysis of the inorganic core generates radicals through metal–carbon bond fission, followed by their self-coupling, which induces strong covalent and weak bonds simultaneously, causing the solubility of nanoparticles [35]. The underlying mechanism in the photolysis of metal nanoparticles predominantly revolves around the generation of radicals through the homolytic cleavage of metal–carbon bonds. Subsequent coupling of these radicals induces the agglomeration and aggregation of nanoparticles, ultimately resulting in a solubility switch.

These nanoparticles offer high etch resistance as they neither oxidize at higher temperatures nor break down under EUV light exposure, and hence, they offer enhanced thermal and chemical stability [13]. Hence, higher inorganic content is usually desired, with a 65–75% by weight proportion of inorganic content and 25–35% organic content [36]. However, as the demand for smaller nanopattern sizes, lower aspect ratios, and reduced LER increases, further reductions in the sizes of nanoparticles are imperative to reduce light scattering. This pursuit underscores the continuous refinement and optimization of nanoparticle-based photoresist materials to meet the evolving requirements of next-generation lithographic processes. These processes can be based on optical lithography or electron beam lithography.

**Figure 5:**
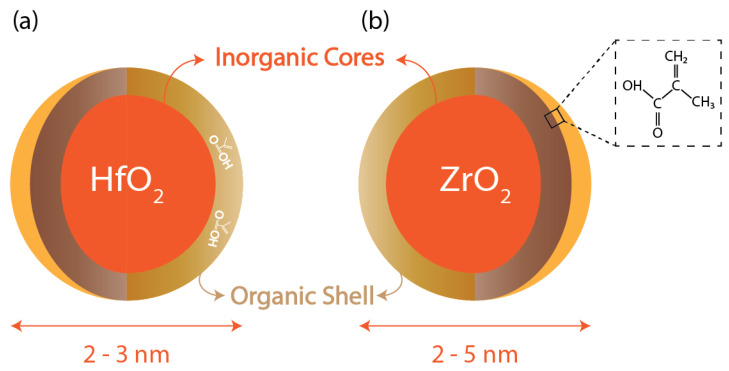
Metal nanoparticle core with organic ligands: (**a**) Hf-oxide core; (**b**) Zr-oxide core with methacrylic acid ligand.

Cornell University research in 2010 reported one of the first metal-oxide-based hybrid resists [36]. This photoresist consists of an inorganic HfO_2_ core, whereas the shell is made of organic ligands, which are electro-reactive, as shown in Figure 5. These resists are dual-tone and, hence, can act as either positive or negative photoresists based on the development conditions. To compare the performance of the resist in positive and negative tones, it was determined under the same experimental conditions and exposure to 254 nm wavelength that the resolutions obtained for positive and negative tones were 0.9 μm and 0.8 μm, respectively [36]. As previously discussed, HfO_2_ offers high chemical and thermal stability and cannot undergo oxidation, unlike organic polymers. Moreover, because of their small size (diameter less than 1 nm), they exhibit minimal light scattering and can be used for both light-based, i.e., DUV and EUV, lithography techniques as well as electron beam ones. This paper reported the results for lithography patterns using these two techniques, and the best resolution achieved was 35 nm using the electron beam lithography technique.

The synthesis of this HfO_2_ nanoparticle can be carried out using two different methods to form precipitates or suspensions in either organic solvents or water [6], and similar techniques can be adopted for the preparation of other nanoparticles.

- Ligand exchange/surface alternation: In this process, carboxylic acid groups replace the surface ligands of the nanoparticles dissolved in an acetate solution after going through heating, precipitation, and washing using acetone steps to get rid of free acid molecules. The product is added to an organic solution to get the desired “hybrid resist”.
- Controlled hydrolysis: Hafnium isopropoxide (C_12_H_28_HfO_4_) undergoes hydrolysis at an elevated temperature for several hours to give HfO_2_ after going through processes such as precipitation, centrifugation, washing, and drying under vacuum. This nanoparticle powder is then added to an organic substance to form the hybrid resist.

Patterning techniques using nanoparticle resists can be carried out using two different mechanisms: ligand displacement and condensation. In the former method, a photoacid generator (PAG) is used that provides photosensitive ligands under exposure, e.g., sulfonate groups, and replaces the ligands of the nanoparticles which have weaker binding to the inorganic/nanoparticle core: hence, changing the solubility of the photoresist portion exposed to the EUV light compared to the unexposed region. This is how sensitivity to EUV light is achieved, and this technique led to 30 nm spacing between lines [37]. In the surface condensation method, no photoacid initiator or generator is used. When the nanoparticle-based hybrid resist is exposed to EUV, the carboxylate groups (COO^−^) become detached from the surface of the resist, resulting in a change in the charge spread and electric double layers, which can generate clusters on the surface; these clusters may even undergo polymerization, but then the resist will become negative-tone.

Similarly, ZrO_2_-based nanoparticles that are capped with a variety of organic ligands can be synthesized through the sol-gel process and utilized for EUV lithography. In this context, a recent study [14] investigated the impact of various ligands on the photochemical conversion of the ZrO_2_ nanoparticle network. The thin film of nanoparticles exhibited a notable reduction in the intensity of the carbon–oxygen bonds of the carboxylic group and resulted in decarboxylation, leading to the release of gaseous CO_2_. This process uses the thiol-ene reaction between multi-alkene-based ZrO_2_ nanoparticles and thiol complexes and allows highly sensitive lithographic nanofabrication.

**Figure 6:**
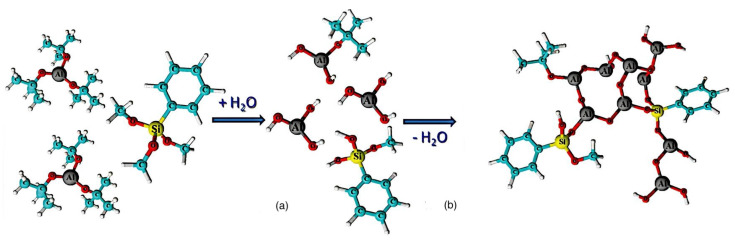
(**a**) Hydrolysis and (**b**) condensation of metal−organic precursors [38].

Following this development, Nam’s group developed another aluminum-core-based organometallic photoresist [28]. After depositing PMMA film to a substrate, they employed atomic layer deposition to incorporate trimethyl aluminum (TMA) into the film. TMA was subsequently transformed to aluminum oxide (AlO_x_) through the addition of steam [38]. The amount of AlO_x_ produced by PMMA could be regulated by altering the number of cycles. With a six-times more effective contrast ratio and a 70-times higher etch resistance selectivity to silicon than regular PMMA, this composite photoresist showed better performance in E-beam lithography. The novel resist system was produced by deploying the method known as sol-gel using aluminum tri-sec-butoxide and a phenyl-modified silane precursor. This spin-on resist is unique because it is coated with a sol composed primarily of an alumina precursor. Following deposition, the precursor reaches an appropriate condensation degree so that it transforms into an organically modified alumina-like film (Figure 6) that lacks the addition of solid fractions, including nanoparticles or nanocrystals. The team led by Grenci and Brusatin also explored aluminum-containing photoresists: adding boehmite nanoparticles to a sol-gel silica-based system that was radiation-sensitive [24].

An aluminum-containing precursor was synthesized utilizing an alumina-like ceramic resist film after two major steps, which included mild thermal treatment and exposure to X-rays. The utilization of extremely intense X-ray radiation for consistent exposure and the impact of residual nanoparticles on the final pattern’s quality might be some viable solutions to address the downsides of resolution restrictions caused by nanoparticle size. An organic–inorganic combination photoresist with a 60:1 selectivity using ICP plasma etching was the final product. Nam’s team also investigated aluminum tri-sec-butoxide and a silane reagent modified with phenyl as thin film precursors for photoresists. They demonstrated that the resist could switch between negative and positive photoresist tones by examining how well it worked in UV and E-beam lithography. The photoresist demonstrated a remarkable 100:1 etch resistance in fluorine-containing plasma etching investigations compared to the underlying silicon [24].

#### 2.3.2. Low Molecular Weight Complexes

These compounds, also referred to as molecular organometallic resists, exhibit sizes smaller than nanoparticles [5] while holding EUV-absorbing metal atoms. These coordination compounds, with their intricate structures composed of numerous metal atoms, offer the potential for high-EUV-absorption cross-sections and increased sensitivity [4]. These molecular organometallics further allow structure-tailoring ability in organometallic synthesis, especially for transition metal complexes at the atomic level [39], allowing for optimal performance in EUV photolithography.

Molecular photoresists have higher solubility, an easy spin-coating process, and higher metal content, which increases the absorption efficiency of these materials. In most cases, these compounds contain metal–carbon bonds capable of undergoing photochemical homolytic bonding to produce radical species. Meanwhile, heteroatoms like nitrogen and oxygen surround the metal center’s remaining chelating sites. Typically, the chelating sites are also occupied by multidentate ligands like phthalocyanine, carboxylate, xanthate, and oxalate.

Sortland et al. in [8] studied the behavior of mononuclear complexes under EUV exposure of transition metals platinum and palladium, including the carbonates L_2_M(CO_3_) and oxalates L_2_M(C_2_O_4_), where M is palladium or platinum here, L is the ligand, and the complex group is carbonate or oxalate. They discovered the first mononuclear organometallic compound in the form of metal oxalates that behaved as positive photoresists. Generally, palladium complexes made photoresists that were faster than platinum ones.

**Figure 7:**
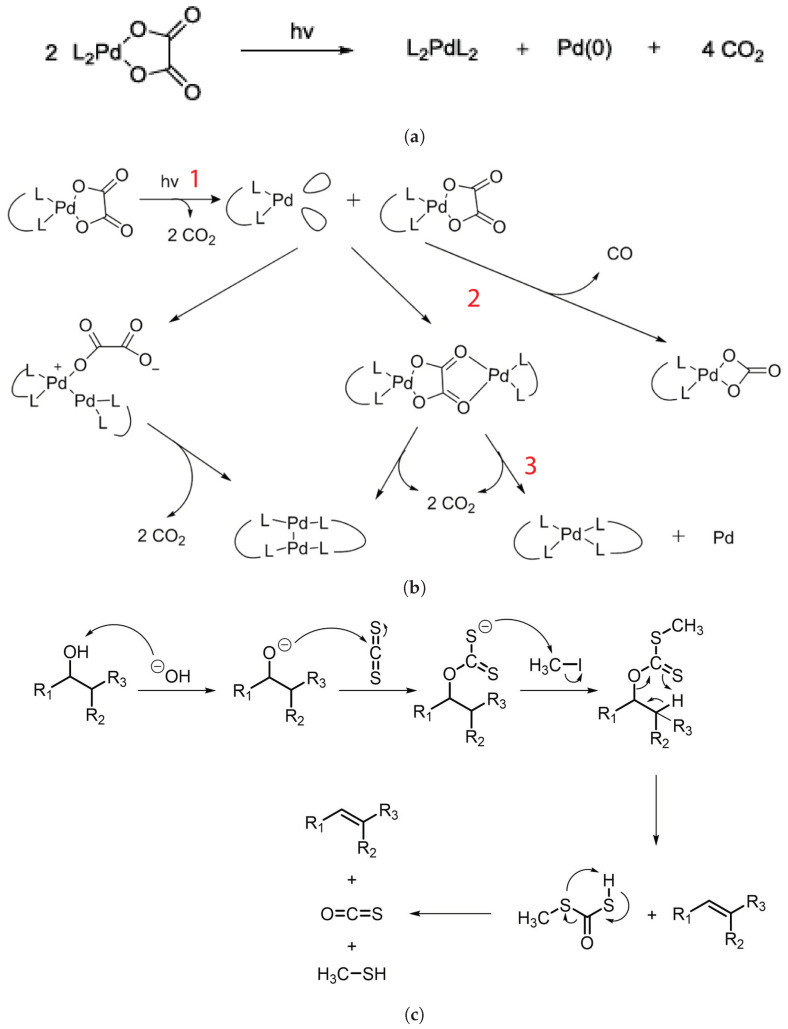
(**a**) Photoreaction of palladium complex. (**b**) Synthesis process of palladium complex photoresist and its intermediate stages [40]. (**c**) Chugaev elimination reaction [4].

Considering the palladium complex to explain the mechanism of the photoresist, it was determined to be mainly caused by palladium oxalates based on experimental data and literature studies [40]. The exposure of L_2_PdC_2_O_4_ to EUV removes two CO_2_ molecules, and a reactive intermediate L_2_Pd is produced, which in turn reacts with another oxalate molecule to ultimately produce a L_2_PdL_2_ complex in addition to one Pd and two molecules of CO_2_. The resulting complex is nonpolar, which results in high solubility in nonpolar developer solution: hence, the “positive” tone resist. The overall reaction is shown in Figure 7a, whereas Figure 7b shows the intermediate stages, with different states marked as 1, 2, and 3.

The synthesis of the most promising palladium complex—dppmPdC_2_O_4_, where dppm = 1,1-bis(diphenylphosphine)methane, which is based on a phosphine ligand and oxalate complex—is done by dissolving the solids in a 1:2 ratio in acetonitrile/ethyl lactate or methylene chloride. The solution is then filtered. Upon spin coating the resist on a silicon wafer, a 40–60 nm-thick resist coating is deposited, and after EUV exposure, it is developed using a combination of MIBK and toluene/hexanes. The resolution achieved using this photoresist is 30 nm-thick lines utilizing light exposure of 50 mJ/cm^2^. For molecular organometals, like metal sulfides, a technique reminiscent of the one used to manufacture these compounds is achieved and patterned using e-beam lithography for feature sizes of sub-10 nm [41].

**Figure 8:**
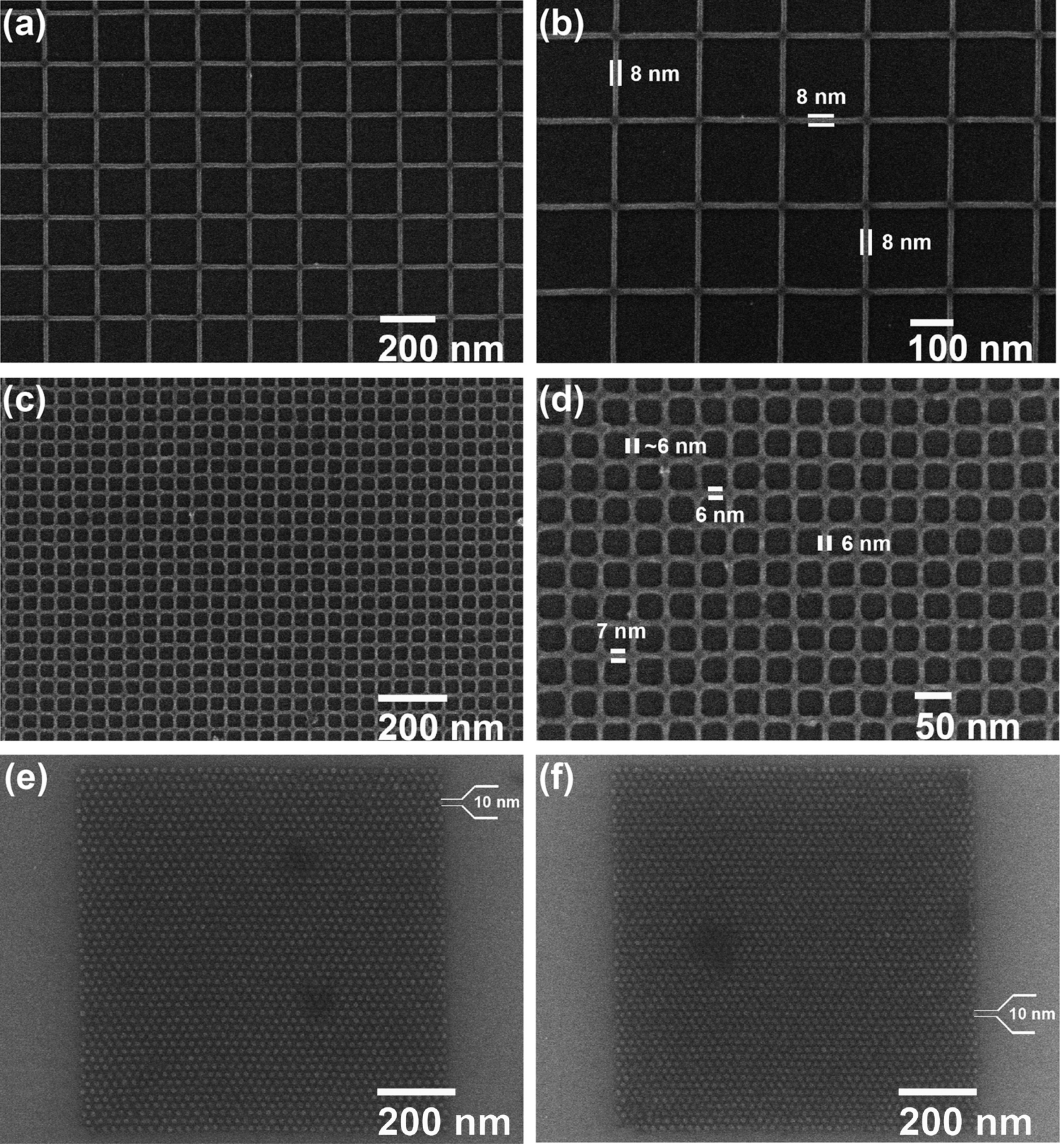
SEM images of (**a**,**b**) 8 nm ZnS lines, (**c**,**d**) 6 nm ZnS lines, and (**e**,**f**) 10 nm ZnS dots with pitches of 25 and 22 nm, respectively [41].

Similarly, the formation of Zn complexes involves the Chugaev elimination reaction (Figure 7c), which reduces the alcohol, followed by a reaction with carbon disulfide (C_2_S), and the xanthate intermediate compound is then trapped with methyl iodide [42]. Consequently, unimolecular elimination removes the HX substituent, resulting in the formation of a double bond. In the case of Zn, the electron beam falls on the Zn^2+^/Cd^2+^ mononuclear complex, which features two pyridines and two alkyl xanthates as ligand derivatives, and it undergoes excitation and loses the pyridine ligands (as in Figure 7c). The resultant complex proceeds through a cyclic transition state, where the hydrogen separates from the β-carbon atom and attaches to the xanthate oxygen, resulting in the sulfide formation of zinc, carbonyl, and hydrogen. Ultimately, ZnS/CdS allows the fabrication of 6 nm-thin metal sulfide lines, and pyridines and alkyl xanthates allow for even smaller features, down to 4 nm, along with 10 nm dots, as in Figure 8.

The achievement of 4 nm feature sizes using low-molecular-weight complexes represents a significant milestone in lithography and semiconductor technology. This allows for unprecedented miniaturization levels and precision in device fabrication, opening the way for the development of advanced SoCs with enhanced performance and functionality. TSMC has pushed it further to 3 nm in the Apple A17 Pro chipset [43], even offering improved affordability and a higher yield rate than the previous-generation chips.

### 2.4. Dry Film Photoresists

**Figure 9:**
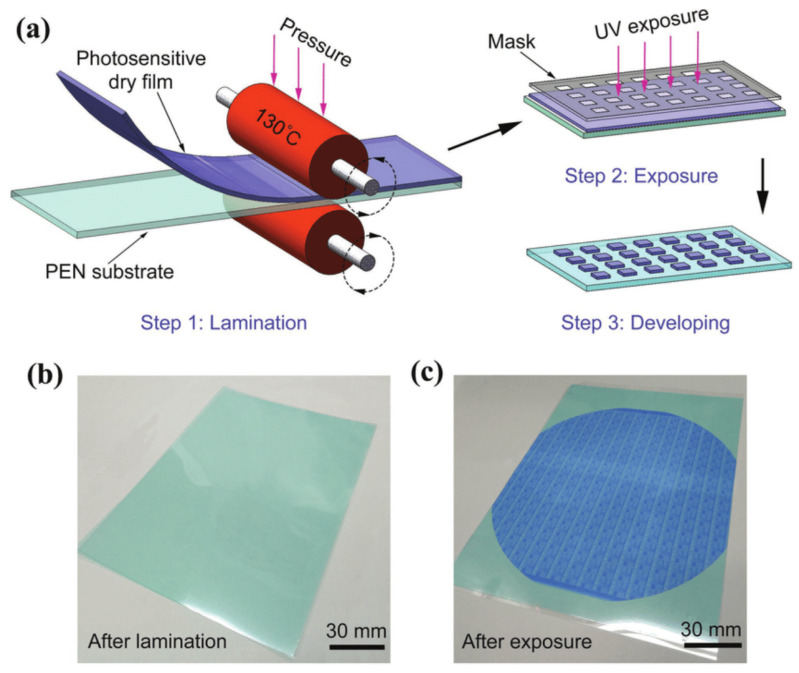
Typical dry film photoresist lamination process demonstrating (**a**) Lamination of a DFP film on a substrate using roll on technique (**b**) The substrate after lamination and (**c**) After exposure with UV light showing the patterns [47].

In recent years, wet film photoresists have been able to achieve under-5 nm nodes, but the uniformity of the resists is still an issue because of the uneven distribution of resists in the spin coating method. An alternative approach to this is the development of dry film photoresists (DFPs), which has advanced significantly in recent years. These are photosensitive polymeric foils that can be directly laminated on wafers, PCBs, etc., and later micropatterned by different lithography techniques [44]. DFPs can either be positive or negative depending on the formulation and the intended application. In [45], the authors reported employing an etch mask technique to utilize dry film photoresists in a positive tone method, enabling the development of micropatterned substrates. It was reported in [46] that positive dry film photoresists offer improved photosensitizing speed, development contrast, resolution, and adhesion quality. This section, however, provides a broader overview of how dry film photoresists are utilized rather than concentrating specifically on their applications as positive or negative photoresists. Generally, in the development process of positive dry film photoresists, the areas exposed to UV light are washed away, leaving behind the unexposed portions of the film. Conversely, with negative dry film photoresists, the exposed sections remain intact after development, while the unexposed areas are removed. As the photosensitive polymer in DFPs undergoes photopolymerization upon UV exposure, it results in cross-linking and the formation of a stable patterned structure [45] (Figure 9). This simplifies processing and reduces the risk of solvent-related defects, resulting in improved pattern fidelity and uniformity.

DFPs are usually three-layer structures that include a polyester support membrane, a layer of resist, and a polyolefin sheet, such as polyethylene, above the resist [48]. The resist is developed on the polyethylene support using a solution. Later, by using polyethylene foil, they are rolled into dry films which are later implanted onto substrates for various applications. During application, this polyethylene (polyolefin) support is removed first, keeping the resist on the polyester support [48]. The base polyester is lifted off with precision and accuracy after the lamination and exposure procedure, keeping only the resist.

In [49], the authors reported creating microcantilevers using a DFP ADEX photoresist. As the DFPs are laminated onto a substrate, which is further processed with lithography, a hot roll laminator is used to implant the film, and the film is baked at 65 °C for 5 min; the photoresist was later developed using exposure to propylene glycol methyl ether acetate for 2 min. In terms of light sensitivity, DFP ADEX can be structured in the wavelength range from violet to ultraviolet (340–425 nm); however, above a certain wavelength of 450 nm, the unexposed resist became transparent, allowing for more than 96 percent light penetration.

Even though DFPs are easier to implement, liquid resists still have some distinct advantages over them, especially in terms of economy and performance. Since the DFP film is spread all across the substrate, leading to extra resist hanging over, it introduces non-utilization of proper spacing of the film which leads to their wastage [48]. The resolution of these resists is also limited by the polyester layer, which introduces some light scattering during exposure, creating a broadening of the image and in turn a loss in resolution, unlike liquid resists, which have better accuracy during exposure [48]. Furthermore, as dry films are implanted, they are also not able to fill imperfections such as holes and scratches on the surface of the substrate, which liquid resists can do.

On the other hand, the lack of processes such as soft-baking and relatively low costs make DFPs more attractive than liquid photoresists. Dry film photoresists are also faster in reaction to UV light compared to liquid resists, and they have an advantage with regard to non-adhesion issues [50]. Additionally, their ability to coat both sides of PCB boards and their preformed nature allows for greater flexibility in substrate choice, including applications with textured or irregular surfaces that may pose challenges for liquid photoresists.

## 3. Conclusions and Future Outlook

The paper provides an overview of the recent advancements in positive photoresists, specifically targeting low-technology nodes for semiconductor chips. During the initial studies after Moore’s law became saturated, development focused on n-CARs as a potential replacement for CARs for patterning technology nodes under 30 nm. Although the n-CARs were able to resolve the issue of acid diffusion, the large molecular sizes of these polymeric resists remained a limitation for LER in sub-20 nm nodes. Following these, hybrid resists, being smaller in size, offer high etch resistance and better thermal and chemical stability, leading to enhanced LER and LWR while maintaining the resolution and sensitivity of the patterning structure. The low-molecular-weight organometallic compounds show faster reaction rates, enhanced etch resistance selectivity, and better performance than organometallic nanoparticles because of their small sizes. Despite the capability of wet film photoresists to achieve sub-10 nm nodes, the uneven distribution of the resist in the spin coating process remains a question. To this end, dry film photoresists offer a solvent-free and pre-formed film that can be laminated directly onto the substrate, providing advantages in handling, processing, and pattern fidelity while maintaining thickness uniformity across the structure. To sum up, these advancements pave the way for further innovations in the field of photolithography and microfabrication, addressing challenges and expanding the capabilities of photoresist techniques.

Future research can enhance nanoparticle resists by using precise synthetic methods to control atomic structure, thus improving film homogeneity for enhanced resolution and sensitivity [51]. Modifying nanoparticle surface ligands can optimize photochemical reactions during EUV irradiation, balancing resolution, sensitivity, and line edge roughness [52]. Additionally, exploring multinuclear complexes and diverse metal atoms can boost photosensitivity, while incorporating functional groups or additives can enhance properties like adhesion, biocompatibility, and stimuli-responsiveness [51]. Research might also focus on designing multifunctional inorganic photoresist materials for simultaneous patterning, sensing, and surface modification [44]. Investigating renewable feedstocks, green solvents, and energy-efficient fabrication techniques [53] could further minimize the environmental impact of photoresist production and disposal. All in all, apart from the highlighted areas, research into beyond EUV (BEUV) lithography using 6 nm wavelengths could drive further innovations. However, this requires extensive research before industrial adoption and mass production [54]. Another revolutionary concept in research is the use of quantum technology in lithography, which leverages quantum mechanics for sub-wavelength resolution. These technologies could steer lithography in a new direction [55] while achieving atomic resolution in semiconductor nodes.

## Data Availability

The original contributions presented in the study are included in the article; further inquiries can be directed to the corresponding author.

## Abbreviations

The following abbreviations are used in this manuscript:

MEMS	Micro-Electromechanical Systems
DUV	Deep Ultraviolet
EUV	Extreme Ultraviolet
CARs	Chemically Amplified Resists
n-CARs	Non-Chemically Amplified Resists
LER	Line Edge Roughness
LWR	Line Width Roughness
PMMA	Poly(methyl methacrylate)
DNQ	Diazonaphthoquinone
DFP	Dry Film Photoresist
PCB	Printed Circuit Board
